# The Predictive Value of Early Behavioural Assessments in Pet Dogs – A Longitudinal Study from Neonates to Adults

**DOI:** 10.1371/journal.pone.0101237

**Published:** 2014-07-08

**Authors:** Stefanie Riemer, Corsin Müller, Zsófia Virányi, Ludwig Huber, Friederike Range

**Affiliations:** 1 Clever Dog Lab, Messerli Research Institute, University of Veterinary Medicine Vienna, Medical University of Vienna and University of Vienna, Vienna, Austria; 2 Department of Cognitive Biology, University of Vienna, Vienna, Austria; Texas Christian University, United States of America

## Abstract

Studies on behavioural development in domestic dogs are of relevance for matching puppies with the right families, identifying predispositions for behavioural problems at an early stage, and predicting suitability for service dog work, police or military service. The literature is, however, inconsistent regarding the predictive value of tests performed during the socialisation period. Additionally, some practitioners use tests with neonates to complement later assessments for selecting puppies as working dogs, but these have not been validated. We here present longitudinal data on a cohort of Border collies, followed up from neonate age until adulthood. A neonate test was conducted with 99 Border collie puppies aged 2–10 days to assess activity, vocalisations when isolated and sucking force. At the age of 40–50 days, 134 puppies (including 93 tested as neonates) were tested in a puppy test at their breeders' homes. All dogs were adopted as pet dogs and 50 of them participated in a behavioural test at the age of 1.5 to 2 years with their owners. Linear mixed models found little correspondence between individuals' behaviour in the neonate, puppy and adult test. Exploratory activity was the only behaviour that was significantly correlated between the puppy and the adult test. We conclude that the predictive validity of early tests for predicting specific behavioural traits in adult pet dogs is limited.

## Introduction

It is now widely accepted that nonhuman animals display consistent behavioural differences comparable to human personalities, and moreover that these differences are functional and of evolutionary significance [1]. However, in contrast to the contention that personality means “behavioural differences that are stable across time and situations”, such behaviour differences are often not as fixed as one might expect [2]. Besides influences of situational factors and salient experiences both early and later in life, developmental factors and age can be expected to have major effects on behaviour, and temporal stability over the short term does not preclude behavioural changes over the long term [2]. It is therefore not surprising that behavioural consistency generally decreases as time between test and re-test increases (reviewed in [2], [3]).

### Behavioural development in humans and nonhuman animals

In humans, personality traits become increasingly more stable with age ([4]; reviewed in [5]). In particular, the rank order of personality features within a cohort (i.e. personality relative to that of other individuals) typically remains stable, while there is a general tendency towards decreases in Neuroticism, Extraversion, and Openness, and small increases in Agreeableness and Conscientiousness with age [6]. Some studies have attempted to make predictions about behavioural predispositions already soon after birth. Although available measurement tools have some shortcomings (moderate internal consistency, low convergent validity, inconsistent findings on concurrent validity; reviewed in [7]), moderate levels of predictive validity of neonate assessments for childhood behaviour have been reported. Among the most predictive traits appear to be levels of irritability or distress, which showed some predictiveness up to the age of 15 months [8], [9], reviewed in [10]. Neonate activity was furthermore correlated with activity and openness to new experiences in 4 to 8-year old children [11]. However, often behavioural consistency seems to be limited to relatively short time intervals. For instance, Worobey & Bladja [9] found that infants' responsivity and activity level were related between 2 weeks and 2 months and between 2 months and 1 year of age, respectively, but not between 2 weeks and 1 year of age. No study seems to have followed up the tested infants' behaviours beyond the childhood years.

Few studies investigated the development of individual behavioural differences from birth in nonhuman animals. In a study on infant macaques and baboons from birth until 5 months of age, several behaviours were significantly correlated between consecutive age blocks of 50 days, but only three (of a possible 33) correlations turned out to be significant across nonconsecutive age blocks [12]. Sussman & Ha [13] report considerable behavioural changes in infant pigtailed macaques between birth and 10 months of age and no relationship of determined temperament traits to behaviour in a novel context. Also, a study on captive wolves found no correlations between neonate and later behaviour [14].

Similarly, assessments of behavioural development from juvenile to adult age in birds [15], fish [16], primates [12], [13], [17], [18], horses [19], [20] and domestic cats [21] yielded mixed results. Some studies support consistency of at least some behavioural traits, while others found no consistency across age or consistency only between adjacent age groups, but not over the longer term, implying a pattern of relative stability or gradual change during development. Furthermore, different traits with a different physiological basis may vary in their ontogeny and consistency [22]. For example, in rhesus macaques (*Macaca mulatta*), confidence was rated as stable at all ages, while ratings for excitability showed no stability until adulthood and those for sociability emerged as significant only after the age of 3 years [17].

### Behavioural development in dogs and validity of puppy tests

Behavioural development in domestic dogs has been investigated for practical reasons such as matching puppies, juvenile or adult dogs with the right families, identifying predispositions for behavioural problems at an early stage, and predicting suitability for service dog work, police or military service. A recent meta-analysis suggested that personality is moderately consistent in younger dogs (<1 year, mean r = 0.30) and older dogs (>1 year, mean r = 0.51; reviewed in [22], but the predictive value of early tests (prior to 3 months of age), as frequently performed for the selection of guide dogs, police or military dogs, was not specially addressed.

Some dog trainers test dog puppies as early as at 1–10 days of age to complement behavioural assessments during the socialisation period for selecting service or working dogs (E. Kersting, pers. comm.); however, these neonate assessments have not been scientifically validated. Moreover, although several studies investigated the predictive value of puppy tests conducted at 6–12 weeks of age, results are inconclusive. For the purpose of this paper we use the term puppy test to denote a sequences of behavioural (sub-)tests performed with young dogs during the socialisation period up to the age of 3 months. Such tests are typically aimed at investigating a variety of behavioural predispositions and often include interactions with unfamiliar people, play, exploration of novel environments or objects, and startle stimuli.

Some studies found a level of predictability of puppy test results for the success of guide dogs and police dogs [23]–[25]; nonetheless, the studies with the largest sample sizes yielded less promising results. Wilsson & Sundgren [26] reported poor correspondence between puppy test results and adult dogs' behaviour and performance as service dogs in a sample of 630 German shepherd dogs. Similarly, Asher et al. [27] followed up 465 dogs assessed in a puppy test and subsequently trained as guide dogs and found low predictability of successful certification. Of the 450 dogs that scored above the proposed cut-off point in the behavioural test, 66% reached certification, compared to 64% in the complete sample. In contrast to success, failure was more accurately predicted by the test, as 14 of the 15 dogs that scored below the cut-off point did not reach certification [27].

Moreover, which combination of subtests is deemed predictive is usually based on an a posteriori selection, and selected tests often differ between studies, although playfulness (fetching a toy or following a rug) emerges as predictive in studies of both guide dogs [23] and police dogs [24], [25]. In contrast to the above studies, which used outcomes (i.e. whether or not the dog became certified) as dependent variables, those studies which investigated direct correlations of behaviour traits in puppies of different ages or between puppies and adults generally did not find much evidence of stability [26], [28], [29]. Beaudet et al. [30] evaluated test-retest performance in 30 puppies at 7 and 16 weeks of age and found no relationship between social behaviour scores within this relatively short time period. Goddard & Beilharz [29] report a low predictive value of tests conducted with 4 to 10-week-old puppies. Fearfulness was the only trait which could be predicted to some degree by the age of 3 months or by a summary score combining subtests from 8 weeks to 3 months [28], [29]. Nonetheless, recognizing that predictability increases with age, the authors recommend waiting until the age of 6 months when selecting dogs for breeding based on the fearfulness trait [28].

Published studies differ in the importance attributed to early environment on shaping later behaviour in dogs. Strandberg et al. [31] report little maternal influence, but a larger influence of litter on personality traits as determined in the Swedish Dog Mentality Assessment. In a behavioural assessment of German shepherd dogs at 15 months of age, two of four traits, ‘Confidence’ and ‘Physical Engagement’ (during play with a tennis ball), were affected by factors such as parity, growth rate, litter size or season of birth whereas no early environmental effects were found on the other two components, ‘Social Engagement’ and ‘Aggression’ [32]. Goddard & Beilharz [33] found little effect of variation in the environment prior to 6 weeks of age on success rate in guide dogs for the blind.

In summary, there are some inconsistencies in the puppy test literature, as well as a lack of longitudinal data on behaviour consistency in pet dogs and on the predictive value of neonate assessments in particular. Therefore the aim of the present study was to perform behavioural tests in pet dogs at three ages – during the neonate period (2–10 days of age), during the socialisation period (40–50 days of age) and as adults (1.5–2 years of age) – and to assess the predictability of later behaviour by early behavioural tests.

In the neonate test, activity and vocalisations during a brief isolation period and sucking force were determined. The puppy test and the adult test both included subtests for 1) exploration in a novel environment, 2) interaction with an unfamiliar experimenter, 3) play, 4) a novel object, and 5) a social conflict situation (three restraint tests in the puppy test and a threatening approach by the experimenter in the adult test). As no published study on assessments of neonate dogs are available, predictions were based on findings from neonate assessments in humans, the coping styles model, and personal experiences (E. Kersting, pers. comm.).

In human children, correlations between neonatal movements and high daytime activity at the age of 4–8 years have been reported [34]. Furthermore the coping styles literature indicates that activity, exploration, aggression and boldness are linked, with proactive individuals scoring higher on all of these than reactive individuals [35], [36]. Therefore a positive correlation between activity in the neonate test and exploratory activity and boldness in the later assessments was predicted. As the degree of irritability in human infants is typically assessed by frequencies and duration of fussing and crying [37], we assumed duration and loudness of vocalisations in the neonate dog puppies to be indicative of irritability. In human infants irritability has been linked to distress to limitations or frustration and forms a negative affectivity factor together with fear [10]. Measures of irritability were found to exhibit relatively high stability over time [9]. Thus we predicted neonate vocalisations to be positively correlated with struggling and flight behaviour during restraint tests in the puppy test and with barking or growling during the threatening approach in the adult test; conversely a negative relationship between neonate vocalisations and latency to react to the threatening approach was predicted. Additionally, the following prediction made by practitioners was put to the test: Sucking force in the neonate test is positively related to motivation and thus playfulness in the puppy and the adult test.

We furthermore predicted that corresponding behaviours would be positively correlated between the puppy and the adult test. To test this, we selected those five subtests from the adult test that matched best with subtests from the puppy test (more subtests were conducted in the adult test with the aim of investigating effects of personality on cognitive performance and age differences in behaviour for different studies). Since effects of litter can be expected due to both genetic and early environmental effects, we tested for litter effects on behaviour in the neonate, puppy and adult tests.

### Ethics statement

All procedures were performed in compliance with the Austrian Federal Act on the Protection of Animals (Animal Protection Act – TSchG, BGBl. I Nr.118/2004) and with the consent by the breeders or owners. According to the Austrian Animal Experiments Act (§ 2, Federal Law Gazette No. 501/1989), such non-invasive behavioural studies are not considered as animal experiments and no special permission for use of animals in such studies is required. For the small number of adult tests performed at the University of Veterinary Medicine, approval by the ethics committee (Ethik- und Tierschutzkommission) of the Veterinary University Vienna was obtained on 19th April 2012. Since the owners were only required to interact with their dogs in their usual manner during the experiments and their behaviour was not analyzed, approval for human experimentation was not necessary.

## Methods

To rule out effects of breed differences in the ontogeny of behaviour [29], [38]–[40], members of a single breed, the Border collie, were included in the study. All tested dogs came from small-scale breeders (with typically 1–2 litters per year) that raised their puppies primarily in the house. We tested 99 puppies from 18 litters in the neonate test (age range: 2–10 days). At the age of 40–50 days, 134 puppies were tested in a puppy test (including 93 puppies tested as neonates). All puppies were subsequently adopted as pet dogs. Fifty of these dogs (29 female, 21 male) were also tested as adults (1.5–2 years of age). Table 1 gives an overview of the subjects. Only three subjects, two males and one female, were neutered during the course of the study (between the age of 6 and 12 months) and thus the data for neutered and intact dogs were pooled.

**Table 1:** Summary of subjects tested in the neonate test, the puppy test and the adult test.

	Age range	Total number of tested dogs	Dogs tested in the neonate test	Dogs tested in the puppy test
**Neonate test**	2–10 days	99		
**Puppy test**	40–50 days	134	93	
**Adult test**	1.5–2 years	50	40	45

### Neonate test

Each puppy was tested individually at the breeder's home following a protocol by Erik Kersting (Hundezentrum Canis Familiaris, Roetgen, Germany, pers. comm.; Table 2). Prior to the test, the mother was separated from the litter for a median of 55 min (range 0–245 min). According to E. Kersting (pers. comm.), puppies should ideally be separated from the mothers for two hours; however breeder compliance was variable and therefore separation time was variable. We tested whether this affected the puppies' behaviour and controlled for this statistically. The puppy was removed from the litter box and placed at the centre of a blanket, which was visually divided into a grid of 16 squares (22.5×22.5 cm). All tests were video-recorded from a set distance (approximately 2 m from the centre of the blanket), and durations of puppies' activity and vocalisations and maximum amplitude of vocalisations were assessed from the videos (Table 2). After two minutes, the experimenter picked up the puppy and tried to elicit the sucking reflex by stimulating the puppy's palate with her finger. Sucking force was determined subjectively but based on an objective scale (Table 2). Experimenters always disinfected their hands prior to handling the puppies.

**Table 2:** Variables measured in the neonate test.

Variables measured		Definition	Cronbach's alpha
**Puppy's behaviour on the blanket**			
Duration of activity		Puppy is moving at least one leg, includes tumbling and backwards movements.	0.92
Number of line crossings		Frequency of crossing a line with the head and both forelegs.	0.82
Number of squares visited		Number of different squares entered with the head and both forelegs.	0.95
Duration of vocalisations		Self-explanatory.	
Max. vocal amplitude		Extracted from the audio stream of a video camera, set at a standardised distance of approximately 2 m from the centre of the blanket (range -50 to -3db) and converted to scores of 1-5.	
	**Amplitude Score**	**Amplitude**	
	1	<20≥−50 or no vocalisation	
	2	≤−15≥−20	
	3	≤−10>−15	
	4	≤−5>−10	
	5	≤−3>−5	
**Test of sucking force**	**Sucking Force Score** *	**Description**	
Max. sucking force	0	Does not take the finger.	
	2	Takes finger, but no sucking.	
	4	Sucking, but hardly holds on to finger when removed.	
	6	Sucking, holds on to finger when removed but no “plop” noise when finger is removed.	
	8	Strong sucking; produces “plop” noise when finger is removed.	
	10	Strong sucking; produces “plop” noise when finger is removed; additionally head moves along as finger is removed.	
	12	Very strong sucking; able to support its own weight by sucking on the experimenter's finger.	

*Intermediate scores (1, 3 etc,) were given in unclear/ambiguous cases.

### Puppy test

As detailed in [41], all tests were carried out in rooms unfamiliar to the puppies at the breeders' homes (only one litter had to be tested in a familiar room because no unfamiliar room was available, so no data was taken in the first part of the test – room exploration). All tests were conducted by the same experimenter (SR), who was unfamiliar to the puppies prior to the test. A cameraman filmed the test for subsequent video analysis. The test, which was originally developed for the selection of service dogs (E. Kersting, pers. comm.), lasted about 20 minutes per puppy and consisted of eleven subtests exposing the puppy to different social and non-social stimuli (see Table 3 for descriptions of the relevant subtests and Table 4 for details on scoring methods; [41]). These form part of a test routinely used for assessing puppies' suitability as service dogs (E. Kersting, pers. comm.).

**Table 3:** Summary of the subtests of the puppy test that were used for analysis.

Subtest	Description	Duration
Exploration	The puppy was allowed to explore the unfamiliar room for two minutes; experimenter, cameraman and breeder remained passive.	60 s
Greeting test	The experimenter crouched down approximately 2.5 m away from the puppy and encouraged it to make contact by calling its name, chatting in a friendly voice or clicking her tongue. When the puppy approached, she petted the puppy and talked to it in a friendly way for 20 seconds. If the puppy did not want to approach within 45 seconds, the subtest was terminated.	60 s
Play	The experimenter tried to engage the puppy in play by wiggling a soft toy in front of it. When the puppy was following and/or trying to grab the toy for at least 10 seconds, she threw it two metres away and vocally encouraged the puppy to return to her with the toy. This was repeated three times.	2–3 min
Back test	The experimenter was sitting on the floor and gently turned the puppy on its back, holding it in this position with both hands while casually looking at the puppy, but not staring at it in a threatening way.	25 s
Vetcheck test	Simulated veterinary examination. The experimenter, sitting on the floor, stroked the puppy's body, touched its paws, looked into its ears and examined its teeth.	30 s
Staring test	The experimenter lifted the puppy up, holding it upright under its armpits, so that she could look directly into its eyes. When the puppy averted its gaze, the experimenter reoriented the puppy and took up eye contact again.	30 s
Novel object test	A battery-powered toy looking like a paper bag, approx. 20×10×5 cm, was placed approx. 2 m away from the puppy to assess its reactions to the novel object's erratic movements.	60 s

**Table 4:** Description of behavioural measurements used in the analysis of the puppy test.

Variable	Type	Measure	Description	Cohen's kappa	Cronbach's alpha
***Exploration***					
Move	Duration	% time	Locomotion (Leg movement followed by body movement. Forwards or backwards movement, coding starts when dog starts to move leg). Does not include moving leg for other purposes e.g. pawing at objects or if dog moves legs but does not change its spatial position.		0.96
Inactive	Duration	% time	Sitting, standing or lying without doing anything else (e.g. exploring). Also includes scratching and shaking.		0.80
Explore	Duration	% time	Puppy's nose is <5 cm from ground or from objects, apparently sniffing, mouthing, manipulating, or scratching objects with the paw.		0.98
***Greeting test***					
Approach	Rating	0	Does not approach the experimenter (10 cm from experimenter's hands) within 45 seconds.	0.71	
		1	Approaches the experimenter within 21–45 seconds after she started calling.		
		2	Approaches the experimenter within 11–20 seconds after she started calling.		
		3	Approaches the experimenter within 10 seconds after she started calling.		
Tail-wagging	Rating	0	Wags tail <30% of interaction time.	0.88	
		1	Wags tail 30–69% of interaction time.		
		2	Wags tail 70% or more of interaction time.		
Jumping up	Absence/	0	Does not jump up or climb into experimenter's lap.	0.70	
	Presence	1	Jumps up or climbs into experimenter's lap.		
***Play***					
Follow toy	Frequency	0–3	Number of times the puppy followed the thrown-away toy (total number of trials: 3).	0.83	
Grab toy	Frequency	0–3	Number of times the puppy followed and grabbed the thrown-away toy (total number of trials: 3).	0.72	
Return with toy	Frequency	0–3	Number of times (out of 3) the puppy brings the toy back to experimenter so she can grab the toy. Puppies that return to within 20 cm of experimenter with the toy and stay there for several seconds but do not bring the toy to experimenter directly, receive half a point.	0.69	
***Back test***					
Struggling	Duration	% time	Quick movements of body, head, and legs. Does not include slow movement of individual limbs or the head. Absolute duration in seconds (precision 0.2 s).		0.95
Vocalising	Duration	% time	Duration of vocalisations. Absolute duration in s (precision 0.2 s).		0.84
***Vetcheck test***					
Flight	Absence/	0	No escape attempt (trying to move away with the whole body while being held – does not include movement with the head to avoid teeth control or walking away when not held).	0.83	
	Presence	1	Escape attempt.		
Interaction	Absence/	0	Mouthing or licking of experimenter's fingers/face for <20% of the time.	1.0	
	Presence	1	Mouthing or licking of experimenter's fingers/face for at least 20% of the time.		
Passive	Absence/	0	Shows interaction or flight behaviour.	1.0	
	Presence	1	Shows neither interaction nor flight behaviour.		
***Staring test***					
Look away	Event	Frequency	Averting gaze (head turn away from the experimenter's face). This is followed by the experimenter reorienting the puppy to look into its eyes again.		0.88
***Novel object test***					
Novel object - Approach	Rating	1	Does not approach to within 20 cm of the novel object within 30 s.	0.67	
		2	Approaches to within 20 cm of the novel object after 5 s.		
		3	Approaches to within 20 cm of the novel object within 5 s.		
Novel object - Tail	Rating	1	Tail mostly low.	0.92	
		2	Tail partly low, partly medium/high.		
		3	Tail mostly medium to high.		
Novel object - Hunt	Absence/	0	Puppy does not ‘hunt’ the novel object (jump at the object with the fore paws and/or bite into it).	0.89	
	Presence	1	Puppy ‘hunts’ the novel object (i.e., jumps at the object with their fore paws and/or bites into it).		
Novel object - Distance	Estimate	continuous	Estimated closest distance (cm) of puppy to paper bag.		0.88

As a measure of interobserver reliability, Cohen's kappa is indicated for scores and Cronbach's alpha for durations, counts, and absolute estimates.

### Adult test

The adult test was specifically designed for use at the Clever Dog Lab with the primary aim of investigating effects of personality on cognitive performance and age differences in behaviour. Partly, the dogs of the current study were used for these other studies and so the test was not completely tailored to serve as a follow up of the puppy test. To take account of this, only the five subtests that matched best with subtests from the puppy test were selected for the present analysis (Tables 5 and 6).

**Table 5:** Summary of the subtests of the adult test that were used for analysis.

Subtest	Description	Duration
Exploration	This was the very first test, conducted in an unfamiliar room. The owner walks in with the dog on the lead, stops in the middle of the room, takes off the lead, gives a “go” command if necessary and thereafter ignores the dog, which is free to explore the room.	120 s
Greeting test	The owner and the dog (on the lead) stand in the centre of the test room. The experimenter enters, steps within reach of the lead, stops and waits whether the dog shows initiative to approach. If it does not, she calls the dog's name and encourages it to approach. If the dog still does not approach, she steps towards the dog. If the dog has approached or does not withdraw, she pets the dog while continually talking to it. If the dog shows avoidance behaviour, petting is stopped.	30 s
Threatening approach	The owner holds the dog's leash but takes one step back so that s/he is behind the dog (giving the dog the opportunity to withdraw behind the owner if it wishes to do so). The owner remains passive throughout the test. The experimenter stands at the opposite end of the room, calls the dog's name once and then starts approaching slowly and haltingly (one step every ∼4 s) with a slightly bent upper body. She is looking steadily into the eyes of the dog. The approach is terminated when the experimenter has reached the dog, the dog has approached the experimenter in a friendly way, or the dog shows heightened signs of stress (repeated barking, growling, or withdrawing/hiding). The experimenter resolves the situation by withdrawing eye contact, crouching down sideways and inviting the dog to come up to her, speaking to the dog in a friendly manner.	30 s
Novel object	A battery-driven toy dog, which rolls on the floor and produces a ‘laughing’ noise is placed on the floor ca. 2 m from the dog while the dog is facing in the other direction with the owner. As soon as the toy starts moving and producing sound, the owner lets go of the dog's collar/harness and the dog has one minute to investigate the toy while owner and experimenter remain passive. The toy is motion sensitive and stops acting after about 15 s. If the dog does not approach close enough to turn the toy on again within 30 s, the experimenter walks past the toy once to turn it on a second time.	60 s
Ball play	The owner throws a tennis ball for the dog three times. During the first two times, the dog is encouraged to bring back the ball. After throwing for the third time, the owner stops interacting with the dog, stands up straight and ignores the dog.	30 s

**Table 6:** Description of behavioural measurements used in the analysis of the adult test.

Variable	Type	Measure	Description	Cohen's kappa	Cronbach's alpha
***Exploration***					
Move	Duration	% time	Locomotion, movement of the legs leading to a forward or backward motion.		0.87
Explore	Duration	% time	The dog's nose is in close proximity (max. 10 cm) to the floor or any other surface (e.g., wall, table, objects) or both front paws placed on an elevated surface (e.g., window sill, table). Does not include drinking.		0.80
Inactive	Duration	% time	Sitting, standing or lying without doing anything else (e.g. exploring). Also includes scratching and shaking.		0.96
***Greeting test***					
Greeting intensity	Score	0	Dog does not approach or may approach initially but then avoid the experimenter so there is no interaction.	0.67	
		1	Dog is passive and shows little interest towards the experimenter, with or without tail wagging		
		2	Friendly greeting; tail wagging, may cuddle up, jump or lick		
		3	Very excited/enthusiastic greeting with intensive searching for contact and tail wagging		
Tail-wagging		0	0 = no or very little wagging	0.71	
		1	1 = wagging intermittently		
		2	2 = wagging most of the time		
Jumping up	Absence/	0	Dog does not jump up in the first greeting phase.	0.82	
	Presence	1	Dog jump ups in the first greeting phase.		
***Threatening approach***					
Latency to react	Latency		Latency to first overt reaction .e. moving away, hiding, barking, growling. This only refers to aversive reactions, but not to approaching the experimenter in a friendly/appeasing way.		0.77
Bark	Absence/Presence	0/1	Absence or presence of barking.	0.89	
Growl	Absence/Presence	0/1	Absence or presence of growling.	0.90	
Retreat	Absence/Presence	0/1	Absence or presence of retreating.	0.89	
Approach friendly	Absence/Presence	0/1	Absence or presence of approaching the experimenter in a friendly/appeasing way during the threatening approach.	0.84	
***Novel object test***					
Novel object - Approach	Score	0	The dog does not approach the novel object to within 20 cm within 60 s.	0.72	
		1	Upon noticing the novel object, the dog approaches to within 20 cm within 60 s.		
		2	Upon noticing the novel object, the dog approaches to within 20 cm within 30 s.		
		3	Upon noticing the novel object, the dog approaches to within 20 cm within 5 sec.		
Novel object - Proximity	Duration		Time spent within 1 m from the toy.		0.97
Novel object - Orientation	Duration		Time spent looking in the direction of the toy		0.94
Novel object - Grab	Absence/	0	The dog does not grab the novel object with its mouth.	0.84	
	Presence	1	The dog grabs the novel object with its mouth.		
***Ball play***					
Return with toy	Frequency	0–3	Number of times the dog returns to within 1.5 m of the owner within 5 seconds of grabbing the ball after it has been thrown.	0.74	
Encourage			Latency to stop encouraging the owner who is ignoring the dog after the third throwing.Encouraging is defined as looking at the owner or spitting out the ball within 1.5 m from the owner while facing the owner.	0.75	
	Score	1	before 5 s		
		2	before 10 s		
		3	before 15 s		
		4	after 15 s		

As a measure of interobserver reliability, Cohen's kappa is indicated for scores and Cronbach's alpha for durations, counts, and absolute estimates.

Tests were conducted in a room (6 m×5 m) at the Clever Dog Lab, Nussgasse, Vienna, or in a slightly larger room (6 m×7 m) with an identical setup at the new Clever Dog Lab, University of Veterinary Medicine, Veterinärplatz, Vienna. Twenty-five dogs were tested by SR and 25 dogs were tested by an another female experimenter of a similar age, Claudia Rosam, as SR had been in contact with many of the tested dogs prior to the adult test. The experimenters were thus unfamiliar to the dogs. An exception were five dogs tested by SR (with four dogs she had had contact at least one year prior to the test, and for one dog the last contact occurred 8 months prior to the test).

### Data processing and statistical analysis

For the neonate test, audio streams were extracted from the video recordings, and the maximum amplitude of the vocalisations was determined in CoolEdit 2000 and subsequently converted into scores of 1–5 (Table 2). The dogs' behaviour in the three tests was coded using Solomon coder (© András Péter). The duration of puppies' vocalisations during the neonate test had to be recorded live during the test because on the video recordings, the subject's vocalisations could not be reliably distinguished from those made by its siblings. The neonate test and the puppy test were coded by the first author. To assess reliability, an additional coder coded 20 randomly selected puppies of 20 litters in the neonate test. Reliability coding for the puppy test was split between two more coders, each of whom coded a subset of the test for 20 puppies. The adult personality tests of the sample presented here, and of an additional 124 dogs tested for other studies, were coded by one of three coders (SR, Stephen Jones, Claudia Rosam). Reliability between coders was assessed based on 38 double coded dogs. Details of the coding schemes and reliability measures are presented in Tables 2–6.

Statistical analysis was carried out in R 2.12.0 (R Development Core Team 2010) and SPSS Statistics 21 (IBM Corp. Armonk, NY, 2012). Non-linear principal components analysis (CATPCA in SPSS [42], [43]) was performed on selected variables from the neonate, the puppy and the adult tests, respectively, to reduce the number of variables and obtain principle components for further analysis. Tables 7–9 show the variable loadings on the principal components, Eigenvalues and explained variance. In the case of the adult test, the sample used for variable reduction included the 50 dogs from the current study and an additional 124 dogs that were tested for other experiments (some of these dogs were tested by a third experimenter).

**Table 7:** Components and component loadings of the CATPCA over the neonate test.

	Activity	Vocal/Sucking force	Total
Activity	0.77	−0.05	
Line crossings	0.83	−0.36	
Squares visited	0.82	−0.39	
Duration of vocalisations	0.49	0.64	
Max. amplitude of vocalisations (score)	0.44	0.66	
Max. suckingforce	−0.08	−0.67	
*Eigenvalue*	*2.38*	*1.57*	*3.95*
*% variance*	*39.69*	*26.17*	*65.86*

**Table 8:** Components and component loadings of CATPCA over selected variables from the puppy test.

**Exploration - Inactivity**		**Greeting**		**Play**		
Move	−0.75	Approach	0.77	Follow toy	0.88	
Explore	−0.86	Tail-wagging	0.82	Grab toy	0.94	
Inactive	0.96	Jumping up	0.68	Return with toy	0.66	
*Eigenvalue*	*2.22*	*Eigenvalue*	*1.74*	*Eigenvalue*	*2.09*	
*% variance*	*73.83*	*% variance*	*57.93*	*% variance*	*69.77*	

Novel object – low Boldness		Restraint tests	Passive/Low Interaction	Play	Struggle	Total
Novel object - Tail	−0.81	Struggling	−0.46	−0.03	0.77	
Novel object - Approach	−0.80	Vocalising	−0.45	−0.42	−0.34	
Novel object - Hunt	−0.72	Flight	−0.25	0.88	0.10	
Novel object - Distance	0.85	Interaction	−0.79	−0.24	−0.37	
		Passive	0.78	−0.53	0.24	
		Look away	−0.59	−0.43	0.41	
*Eigenvalue*	*2.54*	*Eigenvalue*	*2.05*	*1.48*	*1.08*	*4.61*
*% variance*	*63.48*	*% variance*	*34.15*	*24.59*	*18.00*	*76.74*

**Table 9:** Components and component loadings of CATPCA over selected variables from the adult test.

**Exploration - Activity**		**Greeting**		**Play**	
Explore - move	0.90	Greeting intensity	0.90	Encourage	0.82
Explore - explore	0.87	Tail wagging	0.73	Return with toy	0.82
Explore - inactive	−0.85	Jumping up	0.85	*Eigenvalue*	*1.34*
*Eigenvalue*	*2.30*	*Eigenvalue*	*2.06*		
*% variance*	*76.50*	*% variance*	*68.77*	*% variance*	*67.04*

Novel Object - Boldness		Threatening approach	Threat - friendly	Threat - retreat	Total
Novel object - grab	0.92	Latency to react	0.84	−0.20	
Novel Object - approach	0.88	Bark	−0.70	−0.52	
Novel Object - proximity	0.47	Growl	−0.71	−0.53	
Novel Object - orientation	0.56	Retreat	−0.59	0.61	
		Approach friendly	0.63	0.11	
*Eigenvalue*	*2.16*	*Eigenvalue*	*2.64*	*1.36*	*4.01*
*% variance*	*54.04*	*% variance*	*0.33*	*0.17*	*0.50*

Initially, linear mixed models were calculated to assess effects of age, weight and time separated from the mother on the neonate puppies' behaviour, with litter included as a random factor (R package nlme [44], function lme). In case of a significant effect of these covariates, the residuals of the model were used as predictor in subsequent analysis. To assess correlations between earlier and later behaviours, linear mixed models (Type III Sums of Squares) were calculated using either principal components or individual variables, depending on the predictions. To test for litter effects, these models were then compared against models with no random factor included (package nlme [44], function gls). If there was no significant difference according to likelihood ratio tests, the reduced models are presented (Tables 10–12). For variables that were not included as dependent variables in any models, litter effects were calculated in the same way by using likelihood ratio tests to compare models with and without litter as a random factor. Normality of the residuals was assessed from quantile-quantile-plots and was adequate in all cases. To correct for multiple comparisons, sequential Bonferroni correction [45] was applied.

**Table 10:** Summary of linear mixed models testing for predicted associations between neonate test components and puppy test components.

NEONATE test component	PUPPY test variable/component	Predicted direction of effect	Random effect of best model	Value	Std. Error	numDF	denDF	F	P
Activity	Exploration –Inactivity	-	Litter (p<0.0001)	0.06	0.08	1	85	0.52	0.47
Activity	Novel object – Low boldness	-	Litter (p = 0.002)	0.01	0.10	1	85	0.02	0.89
Vocal/Sucking force (residuals)	Flight	+	Litter (p = 0.005)	0.09	0.12	1	74	0.51	0.48
Vocal/Sucking force (residuals)	Struggle	+	Litter (p = 0.0004)	−0.28	0.11	1	74	6.45	0.013
Vocal/Sucking force (residuals)	Playfulness	-	Litter (p = 0.003)	−0.15	0.12	1	74	1.76	0.19

**Table 11:** Summary of linear mixed models testing for predicted associations between neonate test components and adult test components.

NEONATE test component	ADULT test component	Predicted direction of effect	Random effect of best model	Value	Std. Error	numDF	denDF	F	P
Activity	Exploration – High Activity	+	None	−0.15	0.13	1	40	1.41	0.24
Activity	Novel object –Boldness	+	None	−0.16	0.11	1	40	2.23	0.14
Vocal/Sucking force (residuals)	Threat-Retreat (no barking or growling)	-	None	0.12	0.23	1	37	0.26	0.61
Vocal/Sucking force (residuals)	Threat-Friendly (no barking, growling, retreating)	-	None (tendency for litter: p = 0.052)	−0.03	0.22	1	24	0.02	0.88
Vocal/Sucking force (residuals)	Threat reaction latency	-	None	0.10	3.22	1	35	0.001	0.98
Vocal/Sucking force (residuals)	Playfulness	-	None	0.61	0.32	1	35	3.53	0.07

**Table 12:** Summary of linear mixed models testing for predicted associations between puppy test components and adult test components.

PUPPY test component	ADULT test component	Predicted direction of effect	Random effect of best model	Value	Std. Error	numDF	denDF	F	P
Exploration – Low Activity	Exploration – High Activity	-	None	−0.29	0.10	1	43	7.79	0.008
Novel object – Low Boldness	Novel object – High Boldness	-	None	−0.04	0.06	1	43	0.46	0.50
Greeting	Greeting	+	Litter (p = 0.019)	−0.01	0.14	1	29	0.001	0.97
Play	Play	+	None	−0.16	0.18	1	43	0.84	0.37
Passive/Low Interaction	Threat-Retreat (no barking or growling)	+	None	0.07	0.14	1	41	0.10	0.75
Flight		+	None	−0.17	0.14	1	41	1.52	0.23
Struggle		-	None	−0.24	0.14	1	41	2.74	0.11
Passive/Low Interaction	Threat-Friendly (no barking, growling, retreating)	-	None	0.06	0.16	1	24	0.11	0.74
Flight		-	None	−0.01	0.16	1	24	0.002	0.96
Struggle		-	None	−0.07	0.16	1	24	0.18	0.68

## Results

### Data reduction and covariates

The CATPCA of the neonate test yielded two components, labelled Activity and Vocal/Sucking force, which accounted for 65.86% of the variance (Table 7). Activity had high positive loadings for all three variables related to activity, i.e. duration of being active, number of line crossings, and number of squares visited. Vocal/Sucking force had high positive loadings for duration and loudness of vocalisations and a high negative loading for sucking force, reflecting the fact that heavier puppies tended to vocalise more but displayed a lower sucking force (Table S1). The positive effect of puppies' weight on the Vocal/Sucking force component was significant, while there was a significant negative effect of separation time. To take account of this, the residuals of the model for Vocal/Sucking force were used as predictors in the subsequent analysis. Activity was unaffected by age, weight or separation time (Table S1).

Tables 8 and 9 show the results of CATPCA for the puppy and the adult test, respectively. Principal components for activity during room exploration, greeting of the experimenter, play with a human and boldness towards a novel object were extracted for both the puppy and the adult test. Note, however, that the components relating to room exploration and boldness had opposite loadings in the puppy and the adult test so that a negative relationship would be expected between them. Additionally, three components – labelled Flight, Struggle and Passive/Low Interaction – based on the puppies' predominant reactions to the restraint tests were extracted from the puppy test (Table 8; see [41]). From the adult test, two components based on dogs' reactions to the experimenter's threatening approach were determined. The latter were labelled Threat-Friendly and Threat-Retreat due to high loadings of either friendly approach behaviour or withdrawing from the threatening experimenter, respectively (Table 9). Both components had high negative loadings for barking and growling.

### Associations between behaviour in the neonate test, the puppy test and the adult test

Although struggling in the puppy test was negatively associated with the residuals of the Vocal/Sucking force component in the neonate test (F_1,74_ = 6.45, p = 0.013) this effect disappeared after correcting for multiple testing. None of the other tested variables in either the puppy or the adult test was significantly correlated with the predictors from the neonate test (Tables 10–11), indicating a lack of predictive value of the neonate test used. Regarding associations between behaviour in the puppy test at 6–7 weeks and the adult test, only a single significant correlation emerged: as predicted, Exploration - Inactivity in the puppy test was negatively correlated with Exploration - Activity in the adult test (F_1,43_ = 7.79, p = 0.008; significant after correction for multiple testing). None of the other predicted associations turned out to be significant (all p>0.1, Table 12).

### Litter effects

In the neonate test, Activity was unaffected by litter (p = 0.30) whereas Vocal/Sucking force was significantly affected by litter (p = 0.01; Table S1). All tested variables in the puppy test, Exploration - Inactivity (p<0.0001), Low boldness (p = 0.004), Playfulness (p = 0.0008; Table 10), as well as Greeting (p = 0.014), Passive/Low Interaction (p<0.0001), Flight (p = 0.008) and Struggle (p = 0.0003), were significantly affected by litter. In the adult test, only Greeting (p = 0.02), and Threat-Friendly (p =  0.05) tended to be affected by litter, but this was no longer significant when correcting for multiple testing.

## Discussion

We investigated behavioural consistency and the predictive value of early tests in Border collies. The analysis of the neonate test showed that the Vocal/Sucking force component was affected by puppies' weight, as well as by separation time from the mother, and so these factors would need to be taken into account in assessments of neonate puppies. Nonetheless, although we controlled for these effects, there was a lack of correspondence between the behaviour of neonates and the same dogs during the puppy and adult test, implying a lack of validity of this tool for making predictions regarding future behaviour. The results furthermore indicate low predictive validity of the puppy test conducted at 6–7 weeks of age, as activity during room exploration was the only behaviour that was significantly related between the puppy test and the adult test. Even if some of the results became significant at larger sample sizes, this would be of little use to practitioners when assessing individual dogs.

The lack of the predictability of future behaviour based on our neonate test is in line with a study on the ontogeny of behaviour in a litter of captive wolves: MacDonald [14] tested five wolf cubs' reactions to people and novel objects repeatedly from birth to the age of 6 months. He suggests that some consistency in behaviour, relative to the litter mates, did not emerge before the age of 44 days when the cubs were tested together with their siblings. Moreover, in individual tests, individual behaviour differences did not stabilise until day 86. Some major changes were observed over time, with the initially most fearful individuals becoming most friendly to people or vice versa [14]. While these results are in agreement with the lack of correspondence between neonate and later behaviour found in our study, unfortunately the animals were not followed up for more than 6 months and so we do not know whether those individual differences which showed some stability between 6 weeks and 6 months remained stable until adulthood. Also, studies on primates found poor correspondence between behaviour as neonates and 5 to 10 months later: Heath-Lange et al. [12] assessed behaviour of infant macaques and baboons in blocks of 50 days and while several traits were correlated between adjacent age blocks, most behaviours were unrelated over longer time spans [12]. Sussman & Ha [13] report no predictive value of neonate pigtailed macaques' behaviour for later behaviour at all.

In the current study, correspondence between dogs' behaviours at 6–7 weeks and 1.5–2 years was low, with only one out of ten investigated traits being significantly correlated between the puppy and the adult test. This implies that either behaviour is not consistent from the age of 6 weeks or a lack of validity of the assessments used. Given that tests such as those used in the present study are routinely used for selecting working dogs, this is a critical question. Clearly one downside of behavioural assessments in general is that generalisations about the dog's overall behavioural tendencies are made from a test spanning a very limited time period and including a limited number of stimuli [46]. Also, all tests were designed to be appropriate for the respective ages and therefore different assessments were used at different ages. However, it should be considered that the use of different measurements will lead to more diverging results than applying the same instrument twice, confounding the consistency estimate with method variance [22]. These factors may have contributed to the low correspondence between earlier and later behaviour traits in our study.

Another factor that could have contributed to the low consistency is the young age of the puppies in the puppy test. At 6–7 weeks, puppies tend to be quite open and will react less fearfully to stimuli [47] before a heightening of fear responses occurs at around 9-10 weeks of age [48]. Thus, by testing the puppy at 6–7 weeks of age, there was a low risk of detrimental effects on the puppies' socialisation due to the presentation of potentially fear eliciting stimuli such as the novel object (table 4, c.f. [27]). At 6 weeks of age, however, the puppies were only one quarter into their sensitive period which lasts from 4 to 12 weeks of age (sensu Friedman et al. [47]; Lord [49] considers this period to end already at 8 weeks), and later events, particularly environmental influences after transition to their new homes are likely to have had a major influence on the puppies' development. Thus, testing at a later age might have resulted in higher consistency between tests. For instance, when comparing puppies' scores in “fear of object tests” with adult fearfulness, Goddard & Beilharz [29] found no significant correlations between adult fearfulness and behaviour in tests conducted at 6 or 7 weeks of age, but scores in one of three tests conducted at 8 weeks and in two of four tests conducted at 10 weeks were significantly correlated with fearfulness in the adult dogs. Furthermore, trainers' subjective ratings of adult dogs' nervousness, assessed during five different behavioural tests and 3 weeks of training, were significantly positively correlated with “fear on walk” scores at 3, 4, 6 and 12 months of age, respectively, but correlation coefficients increased more than two-fold between 3 and 12 months [28].

While the importance of a sensitive period for socialisation in young puppies is often stressed (e.g. [47], [49]), this does not imply that environmental influences occurring at other developmental stages do not have effects as well [50], and so experiences throughout ontogeny can account for the low correspondence between behaviour in the puppy and the adult test. For example, Appleby et al. [51] found that environmental factors (such as being raised in a nondomestic environment and lack of exposure to urban environments) between the ages of 3 and 6 months were significantly associated with aggressive and avoidance behaviour in pet dogs. Moreover a major reorganisation of the central nervous system occurs during puberty [52], and there is growing evidence that adolescence can be considered as an additional sensitive period (beyond the prenatal and early postnatal periods), with profound effects on future behaviour (reviewed in [53]). There is evidence that steroid-dependent adolescent brain and behavioural development can be modified by social experience [54]. Thus, experiences after the first sensitive period of socialisation, and in particular during adolescence, will also play an important role in determining the adult animal's behaviour. For instance, Foyer et al. [55] point out that the experiences and behaviour of the dogs during their first year of life are crucial in determining their later behaviour and temperament, and accordingly, Swedish military dogs are not selected for enrolment within the Swedish Armed Forces until they are 15–18 months old [55].

A reason for the diverging results of previous studies regarding the predictive value of puppy tests may lie in different levels of analysis. Based on the existing puppy test literature, we suggest that the predictive value of a puppy test depends on the level at which a prediction is made: puppy tests may have the potential of predicting outcomes (successful qualification as guide dogs [23], [28] or as police dogs [24], [25]) to some extent (but see [26], [27]), but not individual behaviour traits [30], [56], [57]. Based on psychometric principles, a higher reliability can be expected for aggregate measures (i.e., sum or average of multiple observed behaviours) than for single measures due to evening out of the random, nonsystematic errors in the different multiple measures [22]. Although there is some evidence that aggregate measures are more predictive of outcomes [58] and have higher heritability estimates [57] than single measures in dog personality assessments, a meta-analysis on personality consistency in dogs did not find a significant difference between single trait measures and aggregate trait measures [22]. At least in the case of puppy tests, however, the current literature seems to support higher predictability for outcomes (i.e. aggregate measures) than for individual behaviour traits, and accordingly, our results show that correlations between puppies' and adults' behaviour are mostly lacking.

Litter effects differed between assessments at different ages. Vocal/Sucking force in the neonate test and all puppy test components were significantly affected by litter whereas in the adult test no significant litter effects were found. This indicates that behaviour in the 6–7-week-old puppies was influenced more by either genetic effects, maternal effects or the shared early environment than behaviour in the adult dogs. Accordingly, high maternal effects are often found in puppies' behaviour but for older dogs, these effects are small or negligible (reviewed in [29]). Studies on other species also showed that effects of early experiences became less salient as the animals became older (e.g. sheep [61]; rats [62]). A decline in the effects of early shared environment with age has furthermore been shown in humans: In more than 200 pairs of adoptive siblings, correlations in IQ of 0.26 were found when the children were 8 years old; however, 10 years later these same siblings showed a correlation near 0.0 [63].

Unlike this study, Strandberg et al. [31] did find litter effects (as well as additive genetic effects) on adult dogs' behaviour in behavioural assessments, and also Foyer et al. [32] identified influences of several early environmental variables on the behaviour of dogs tested at approximately 17 months of age. A possible explanation lies in the bigger sample sizes in these studies (N = 5959 and N = 503, respectively), so that much smaller effect sizes are significant. Heritability of behavioural traits has been estimated at 0.05–0.56 in domestic dogs [59], [60], although there appears to be breed-specific variation [26], [60]. In general, heritabilities around 0.20 appear to be the norm. This effect may be too small to turn out as significant with our sample size and may explain the scarcity of litter effects in the adult test. Thus, the absence of litter effects in our study does not necessarily imply that genetics or early environmental influences are unimportant but indicates that litter effects were too small to be detected in our sample. Conversely, the results point to the importance of (later) environmental influences on canine behaviour.

Furthermore, environmental differences can be expected to have a greater effect on behavioural variability in our sample of pet dogs compared to the working dogs of previous studies, which tend to be kept under more uniform conditions and follow standardised training regimes. Given that dogs are highly responsive to their social environment [64], the role of the owner should not be forgotten. For example, parallels in personality dimensions in humans and their dogs have been reported [65], training methods employed by the owners were found to be related to dogs' openness towards an unfamiliar person and how they interacted with their owners in play [66], and owner personality was related to stress coping in human-dog dyads [67].

### Conclusions

Our results suggest that early behavioural tests yield poor predictability regarding future behaviour in pet dogs. While there are some indications that puppy tests may have the potential to identify negative extremes (e.g. [27]) and may serve to predict outcomes such as working dog success, we want to caution against over-interpreting results from these early assessments and highlight the importance of experiential factors in the course of ontogeny in influencing the adult dog's behaviour. Despite the blossoming of dog research in the last decades, we are still at the beginning of understanding dogs' behavioural development. Future studies should investigate developmental trajectories by repeatedly assessing dogs between the age of 6 weeks and 1.5 years and by following them up into old age. This will yield further insights into the ontogeny of behaviour in dogs and the question from what age meaningful predictions about later behaviour can be made.

## Supporting Information

Table S1 **Final reduced models of effects of age, separation time and weight on the components Activity and Vigour of the neonate tests.** (effects of the interaction between predictors and age are not shown because they were removed in the model selection process).
(DOC)
