# Supplementary material for: The Predictive Value of Early Behavioural Assessments in Pet Dogs – A Longitudinal Study from Neonates to Adults
Source: PLoS One. 2014 Jul 8;9(7):e101237. doi: 10.1371/journal.pone.0101237 (PMC4086890; doi:10.1371/journal.pone.0101237)
Supplement: Table S1 — Final reduced models of effects of age, separation time and weight on the components Activity and Vigour of the neonate tests. (effects of the interaction between predictors and age are not shown because they were removed in the model selection process). (DOC) [file pone.0101237.s001.doc]

**Table S1.**

| **Dependent variable (model)** | **Predictor** | **Random effect of best model^*^** | **Value** | **Std. Error** | **numDF** | **numDF** | **F** | **P** |
| --- | --- | --- | --- | --- | --- | --- | --- | --- |
| Vocal/ Sucking force | Weight | Litter (p=0.003) | 6.13 | 0.02 | 1 | 79 | 0.002 | 0.0008 |
|  | Time separated |  | 6.03 | 0.02 | 1 | 79 | -0.005 | 0.002 |
| Activity | Weight | None | 0.00007 | 0.001 | 1 | 34 | 0.003 | 0.95 |
|  | Time separated |  | 0.003 | 0.003 | 1 | 34 | 0.84 | 0.36 |
